# The V_2_ receptor antagonist tolvaptan counteracts proliferation and invasivity in human cancer cells

**DOI:** 10.1007/s40618-022-01807-5

**Published:** 2022-05-23

**Authors:** G. Marroncini, C. Anceschi, L. Naldi, B. Fibbi, F. Baldanzi, M. Maggi, A. Peri

**Affiliations:** 1 Pituitary Diseases and Sodium Alterations Unit, AOU Careggi, 50139 Florence, Italy; 2 Endocrinology, Department of Experimental and Clinical Biomedical Sciences “Mario Serio”, University of Florence, AOU Careggi, Viale Pieraccini, 6, 50139 Florence, Italy

**Keywords:** Tolvaptan, AVP receptors, Hyponatremia, Cancer, SIAD

## Abstract

**Purpose:**

Hyponatremia, the most frequent electrolyte alteration in clinical practice, has been associated with a worse prognosis in cancer patients. On the other hand, a better outcome has been related to serum sodium normalization. In vitro studies have shown that low extracellular sodium promotes cancer cells proliferation and invasiveness. Tolvaptan, a selective vasopressin receptor type 2 (V_2_) antagonist, has been effectively used in the last decade for the treatment of hyponatremia secondary to the Syndrome of Inappropriate Antidiuresis. A few in vitro data suggested a direct role of tolvaptan in counteracting cancer progression, so far. The aim of this study was to evaluate the effect and the mechanism of action of tolvaptan in cell lines from different tumours [i.e. colon cancer (HCT-8), hepatocarcinoma (HepG2), neuroblastoma (SK-N-AS)].

**Methods and results:**

First, we showed that these cell lines express the V_2_ receptor. Tolvaptan significantly reduced cell proliferation with an IC_50_ in the micromolar range. Accordingly, reduced levels of cAMP, of the catalytic α subunit of PKA, and a reduced pAKT/AKT ratio were observed. Tolvaptan effectively inhibited cell cycle progression, whereas it induced apoptotis. Furthermore, it reduced cell invasiveness. In particular, anchorage-independent growth and the activity of collagenases type IV were blunted in the three cell lines. Accordingly, tolvaptan counteracted the RhoA/ROCK1–2 pathway, which has a pivotal role in regulating cell movement.

**Conclusions:**

Overall, these findings indicate that tolvaptan effectively inhibits tumour progression in vitro. Further studies should clarify whether the V_2_ receptor might be considered a possible target in anti-cancer strategies in the future.

## Introduction

Hyponatraemia is the most frequent electrolyte imbalance in cancer and it occurs in up to 40% of patients at the time of hospital admission [1, 2].

The syndrome of inappropriate antidiuresis (SIAD) is the most frequent cause of hyponatremia in cancer patients [3]. In addition to ectopic vasopressin (AVP) secretion by tumoural cells, particularly in lung cancer [4], other factors may cause SIAD in this setting as follows: several anticancer drugs, including chemotherapeutic agents, molecular targeted agents and immune checkpoint inhibitors, palliative treatments (e.g. opioids), nausea, and pain. Intravenous hydration during chemotherapy or comorbidities associated with hyponatremia (e.g. heart failure, renal failure, liver cirrhosis) may also facilitate serum sodium concentration ([Na^+^]) reduction [4–7]*.*

It is now well known that hyponatremia represents a negative prognostic factor among cancer patients. Indeed, low [Na^+^] has been associated with a significantly decreased progression-free and overall survival in patients with tumours of different organs [1, 8–18]. Furthermore, hyponatremia has been related to increased length of hospital stay and health costs [8, 19–22]. Interestingly, it has been shown that [Na^+^] correction is associated with a reduced mortality [9, 23, 24]. As a matter of fact, serum [Na^+^] has been proposed as a possible biomarker to identify high-risk cancer patients [25].

A crucial question is whether hyponatraemia is a surrogate marker of disease severity or a factor that directly contributes to cancer progression [26]. Admittedly, a clear-cut answer to this question does not exist, yet. However, we have recently demonstrated that low extracellular [Na^+^] promotes cell proliferation, invasion and tumourigenicity in vitro, in different cancer cell lines [i.e. pancreatic adenocarcinoma (PANC-1), neuroblastoma (SK-N-AS, SH-SY5Y), colorectal adenocarcinoma (HCT-8), chronic myeloid leukemia (K562) cells] [27].

The past decade has seen the introduction of a new class of drugs, namely vaptans, which are non-peptide vasopressin receptors antagonists. In particular, a selective antagonist of the AVP receptor type 2 (V_2_), namely tolvaptan, has been approved in 2009 in the U.S. and in Europe for the treatment of adult patients with hyponatremia secondary to SIAD. Tolvaptan induces water diuresis and has been shown to effectively correct hyponatremia in different clinical settings, including cancer patients [28–33]. In view of the aforementioned beneficial effects of the correction of hyponatremia in cancer patients, it is conceivable that tolvaptan may have a favourable effect, too.

In addition, it has to be considered that tolvaptan has been subsequently approved also for the treatment of autosomal dominant polycistic kidney disease (ADPKD) Here, tolvaptan is able to reduce renal cyst growths and the rate of estimated glomerular filtration decrease [34, 35]. This unpredicted effect has been related to its inhibitory effect on intracellular cAMP production in kidney epithelial cells [36]. Noteworthy, in vitro studies have shown that tolvaptan reduces proliferation and invasion in hepatocarcinoma and renal carcinoma cells [37, 38]. Furthermore, very recently we have demonstrated that in small-cell lung cancer cells tolvaptan counteracts cell proliferation and invasivity, yet triggers apoptosis, mainly via the inhibition of the RhoA/ROCK pathway [39].

The aim of the present study was to investigate on the effects and the mechanism of action of tolvaptan in different cell lines from colon cancer, hepatocarcinoma and neuroblastoma.

## Materials and methods

### Cell cultures

Hepatocellular carcinoma (HepG2) (HB-8065, RRID:CVCL_0027), stromal (S)-type (SK-N-AS) neuroblastoma (CRL-2137, RRID:CVCL_1700), and colon carcinoma (HCT-8) (CCL-244, RRID:CVCL_2514) human cell lines were purchased from the American Type Culture Collection (Manassas, VA, USA). Stock cell lines were routinely cultured in Dulbecco's modified Eagle medium or RPMI-1640 supplemented with 10% fetal bovine serum (FBS), L-glutamine and antibiotics (50 U/mL penicillin and 50 μg/mL streptomycin) and maintained at 37 °C in a humidified atmosphere (5% CO_2_/95% air). All the reagents for cell culture were purchased from Sigma Chemical Co. (St. Louis, MO, USA). To analyse the effects of tolvaptan, cells were cultured in DMEM or RPMI medium for 48 h before experiments were performed, as previously described [39].

### Immunofluorescence

Cells were fixed in 4% paraformaldehyde, blocked in PBS/0.1% BSA/0.05% Tween for 1 h, and then incubated with anti-rabbit polyclonal AVPR2 antibody (PA5-75,409, RRID:AB_2719137, Thermo Fisher Scientific, Walthan, MA, USA) (1:100 dilution) and Hoescht 333,342 (NucBlue Live cell Stain Ready Probes reagent, R37605, Life Technologies, Carlsbad, CA, USA) overnight. The day after, cells were washed twice in PBS and stained with anti-Rabbit IgG Alexa Fluor488 secondary antibody for 1 h at room temperature in the dark. Finally, cells were washed and mounted in fluorescent Prolong Gold Antifade medium (P36930, Life Technologies, Carlsbad, CA, USA) for observation under an inverted fluorescence microscope (Leica Biosystems, Milan, Italy) equipped with a camera.

### Analysis of cell proliferation and viability

After 48 h of tolvaptan treatment, tumour cells were harvested by trypsinization and counted twice using a hemocytometer. Cell viability and proliferation were assessed using Cell Counting Kit-8 (Dojindo Molecular Technologies, Rockville, USA). 10.000 cells/well were seeded in 96-well plates and treated with WST-8, which is directly reduced by intracellular dehydrogenases. Once metabolized, WST-8 creates a coloured product (formazan), directly proportional to the number of metabolic and proliferative cells present in wells. The experiments were run according to manufacturer’s protocol, and luminescence (450 nm and 570 nm filter respectively) was recorded with a Wallac multiplate reader (Perkin-Elmer, Monza, Italy). The results were expressed as optical density OD/well (*mean* ± *SE*) normalized versus control. The experiments were performed in 8-wells/sample and at least two times.

### Western blot analysis

Cells were lysed in RIPA lysis buffer supplemented with complete protease and phosphatase inhibitor cocktail, and the protein concentrations were determined using a Bradford protein assay. Cell lysates (20–40 μg of proteins) were fractionated by 12% Mini-PROTEAN TGX Stain-Free Precast Gels (Biorad, Hercules, CA, USA) and transferred onto PVDF membrane (Immobilon, Billerica, Millipore, MA, USA). After 1 h of 5% milk blocking, membranes were incubated with specific primary antibodies: rabbit monoclonal anti-Cofilin (5175, RRID:AB_10622000, Cell signalling Technology, Danvers, MA, USA)(1:1000 dilution), rabbit monoclonal anti-phospho-Cofilin (Ser3) (3313, RRID:AB_2080597, Cell Signaling Technology, Danvers, MA, USA)(1:1000 dilution), rabbit monoclonal anti-HMOX-1 (ab52947, RRID: AB_880536, Abcam, Cambridge, UK)(1:1000 dilution), rabbit monoclonal anti-RhoA (2117, RRID:AB_10693922, Cell Signaling Technology, Danvers, MA, USA)(1:1000 dilution), rabbit polyclonal anti-AVPR2 (PA5-75,409, RRID:AB_2719137, Thermo Fisher Scientific, Walthan, MA, USA) (1:1000 dilution), rabbit monoclonal anti-AKT (SAB4500800, RRID:AB_10742608, Sigma-Aldrich, Saint Louis, MO, USA)(1:1000 dilution), mouse monoclonal anti-phospho-AKT (05–1003, RRID:AB_1586879, Millipore, Burlington, MA, USA)(1:500 dilution), rabbit polyclonal anti-PKA (PA5-17,626, RRID:AB_10985417, Thermo Fisher Scientific, Walthan, MA, USA)(1:1000 dilution). Primary antibodies were incubated overnight at  4 °C, subsequently membranes were washed twice using PBS 1X  and incubated with the specific secondary antibody conjugated to horseradish peroxidase (7076, RRID:AB_330924 or 7074, RRID:AB_2099233, Cell Signaling Technology, Danvers, MA, USA). Chemiluminescent images were acquired with a Bio-Rad ChemiDoc Imaging System (Bio-Rad, Hercules, CA, USA) and through ImageJ Software. Proteins of interest were quantified and normalized versus stain free acquisition.

### Enzyme-linked immunosorbent assay (ELISA)

The intracellular cAMP concentration was measured with the competitive cAMP ELISA Kit (#4339, Cyclic AMP XP® Assay Kit, Cell Signalling Technology) according to the manufacturer's protocol. HCT-8, HepG2 and SK-N-AS cells were cultured in 6-well plates and treated with 30, 50 and 70 μM of tolvaptan (HCT-8 and HepG2) or with 20, 40 and 50 μM of tolvaptan (SK-N-AS). Then, 2.5 × 10^4^ cells for HCT-8 and 5 × 10^4^ cells for HepG2 and SK-NA-S were plated into a 96-well plate. After 24 h, cells were incubated in serum-free medium and subsequently lysed on ice with 100 μl of lysis buffer for 5–10 min and centrifuged at 12,000 rpm for 10 min. Afterwards, the HRP-linked cAMP solution and 1:1 volume of sample was added to the cAMP assay plate and incubated for 3 h. After being washed four times with Wash Buffer 1X, the substrate was added to the assay plate and incubated for 30 min. Following the addition of the stop solution, the absorbance was detected at 450 nm using a microplate reader and the concentration of cAMP was calculated according to a standard curve.

### Immunocytochemistry

HCT-8, SK-N-AS and HepG2 cells were seeded on a glass slide. One day after, tolvaptan was added to the medium and incubated for 48 h before proceeding with the immunostaining, which involves fixation, permeabilization, and antibody incubation. Indeed, cells were first fixed with 4% neutral buffered formalin for 20 min, washed twice in PBS 1X and permeabilized with 0,05% of Triton X-100 for 1 h at room temperature. Afterwards, glass slides were incubated with anti-Cleaved caspase-3 (Asp175) primary antibody (9661, RRID:AB_2341188, Cell Signaling Technology, Danvers, MA, USA) (1:1000 dilution) overnight at 4 °C, and subsequently with anti-rabbit IgG HRP conjugated (7074, RRID:AB_2099233, Cell Signaling Technology, Danvers, MA, USA). Protein were detected using AEC (3-amino-9-ethylcarbazole) Substrate Peroxidase (HRP) Kit, (SK-4200, Vector Laboratories, Burlingame, CA, USA) that create a red precipitate in presence of caspase-3 protein. Slides were counterstained with Gill’s haematoxylin n°3 (05-06015E, Bio Optica Milano Spa, Milan, Italy) and the staining effect was observed and photographed under a microscope. Both control and tolvaptan-treated cells were examined: five fields per slide were randomly chosen under light microscopy at high-power field (× 40) and caspase-3 positive cells have been counted.

### Cell cycle and annexin V/PI analyses

HCT-8, SK-N-AS and HepG2 cells were incubated with tolvaptan for 48 h, harvested and washed twice with sterile PBS to perform cytofluorimetric analysis of cell cycle and annexin V/PI using the Guava® Muse® Cell Analyser (Luminex Corporation, Austin, TX, USA). As previously described, cells must be fixed using 70% ethanol and stored at -20 °C for at least 3 h. After fixing and washing, 1 × 10^6^ and 25 × 10^4^ cells were stained with 200 μl and 100 μl of Muse™ Cell Cycle and Annexin V & Dead Cell Reagent (Luminex Corporation, Austin, TX, USA) respectively, and incubated for 30 min at room temperature before performing cytofluorimetric analysis. Results of cell cycle were expressed as % of cells in G0/G1, S and G2/ M phases; results of annexin V/PI were expressed as % of live and total apoptotic cells normalized versus control cells.

### Invasion analysis and ECM degradation

Invasive migration capacity of cancer cell lines was assessed by a standard Transwell invasion system and by zymography assay. First, 1 × 10^6^ tolvaptan pre-treated tumour cells were induced to migrate under chemoattractant stimuli (FBS serum), using polycarbonate filter inserts (8 μm) (Corning, New York, USA) precoated with 0.3% BD Matrigel Basement Membrane (BD Becton, Dickinson and Company, New Jersey, USA). Finally, 24 h after the induction at 37 °C, invasive cells were stained with 0.1% crystal violet/methanol and observed under the microscope. To obtain a quantitative analysis of migrated cells, the inserts were decoloured with dimethyl sulfoxide (DMSO) and the dye mixture was measured by spectrophotometer (Perkin-Elmer, Monza, Italy) at the optical density (OD) of 560 nm.

After 48 h of tolvaptan exposition, cancer cells supernatant was collected to perform zymography and analysis of type IV collagenases (MMPs) secretion. Samples were separated with Protean II system (Bio-Rad, Hercules, CA, USA), in SDS-PAGE Tris–glycine 8.0% polyacrylamide gel containing 2 mg/ml of gelatine at a constant current of 90 mV. After run, gels were washed twice in 2.5% Triton X-100 for 15 min and incubated in a rocking platform with reaction buffer (50 mM Tris–HCl, pH 7.5, 5 mM CaCl2, 200 mM NaCl and 1% Triton X-100) overnight at 37 °C. The day after, gels were stained with 0.1% Coomassie blue R-250 (Amersham Pharmacia Biotech, Milan, Italy) for at least 1 h in 40% 2-propanol. White bands corresponding to MMP2 and MMP9 were quantified as % of pixels normalized versus untreated cells.

### Soft agar assay

Soft agar assay was used in order to analyse the effect of tolvaptan on anchorage independent growth of tumour cells by preparing a multilayer plate, as previously described [27]. 5 × 10^4^ cells in RPMI 1640 double added with FBS were plated in the upper layer (0.3% of noble agar) together with three different concentrations of tolvaptan (3 wells per group). Cells were maintained at a condition of 37 °C and 5% CO_2_ for 21 days; finally, the number of colonies in each well was counted by using a light microscope after staining with 0.1% crystal violet solution.

### RNA extraction, reverse transcription and qPCR

Total RNA was extracted by using Tri Reagent with DNAse treatment, according to the manufacturer’s instruction, and samples were quantified spectrophotometrically. After reverse transcription (Taqman Reverse Transcription Reagents, Applied Biosystem Inc., Foster City, CA, USA), cDNA was amplified using a Termocycler (Perkin-Elmer Cetus, Norwalk, CT, USA) through three steps (35 cycles): 94 °C for 45 s., 60 °C for 45 s., 72 °C for 60 s. The expression of target genes was quantitatively analysed by real-time RT-PCR using Pre-developed TaqMan® Assays. The probes were Assay-On-Demand products (Applied Biosystem Inc., Foster City, CA, USA) for ROCK-1 (Hs 00178463_m1), ROCK-2 (Hs00153074_m1) and RHOA, (Hs00357608_m1). The mRNA quantitation was based on the comparative Ct (for cycle threshold) method and normalized to 18S RNA (Hs03003631_g1). Results were expressed as ΔΔct normalized versus control sample. All measurements were carried out in triplicate and at least two independent experiments were performed.

### Statistical analysis

All statistical analyses were carried out with the use of SPSS software Version 13.0 (SPSS, Chicago, IL, USA). Data were expressed as the mean ± standard error from at least three separate experiments. Statistical significance of the results was analysed by paired *t*-test, and *P* < 0.05 was considered to be statistically significant.

## Results

### Cell proliferation

**Fig. 1:**
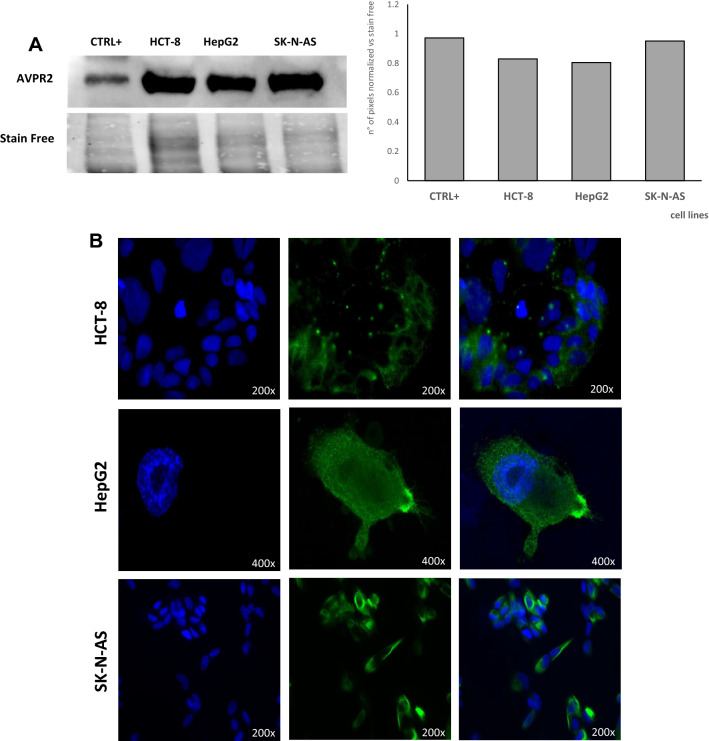
AVP V_2_ receptor expression and localization in HCT-8, HepG2 and SK-N-AS cell lines. **A** Western Blot analysis for V_2_ receptor. Human kidney cells were used as the positive control (CTRL+); **B** V_2_ receptor localization, as evaluated by immunofluorescence staining (green). The nuclei are indicated by the blue color (NucBlue)

First, the expression of the V_2_ receptor in cell lines was assessed. Western blot analysis confirmed that the receptor was expressed in HCT-8, HepG2 and SK-N-AS cells (Fig. 1A). Immunofluorescence indicated the membrane localization of the receptor (Fig. 1B).

**Fig. 2:**
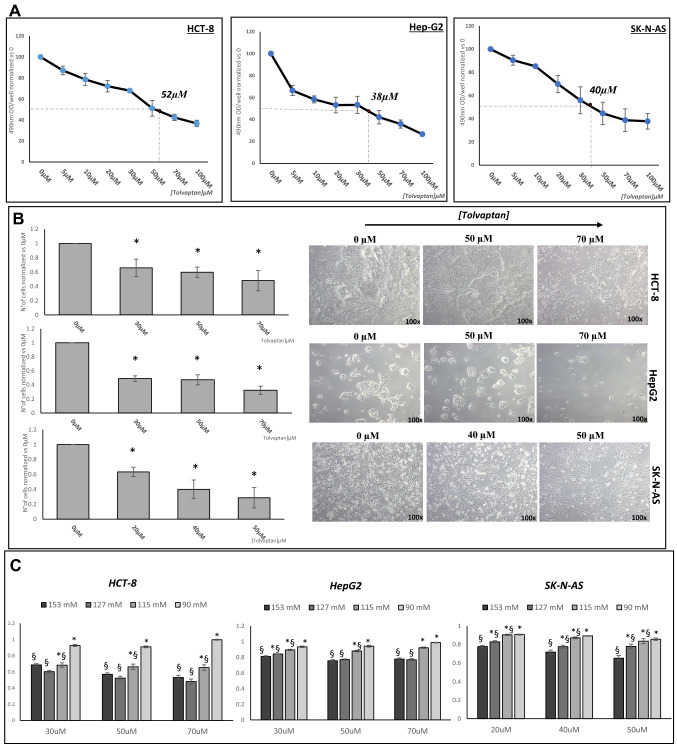
Effect of tolvaptan on HCT8, HepG2 and SK-N-AS cell proliferation. **A**–**B** Dose–response curve of the effect of tolvaptan on proliferation, as assessed by WST-8 assay and identification of the IC_50_ dose. Results are expressed as number of cells ± SE normalized *vs* 0 µM tolvaptan (*n* = 3) (*p ≤ 0.02). Images are representative of cells growth. **C** Effect of tolvaptan on cell proliferation at different [Na^+^], as assessed by WST-8. Results are expressed as number of cells ± SE normalized *vs* 153 mM [Na^+^] (*n* = 3) (*p ≤ 0.05) and *vs* 0 µM tolvaptan (considered as 1) (n = 3) (§p ≤ 0.05)

We found that tolvaptan dose-dependently reduced the rate of cell proliferation of HCT-8, HepG2 and SK-NAS cells, with an IC_50_ of 52 µM, 38 µM and 40 µM, respectively, after a 48 h exposure (Fig. 2A–B). Interestingly, the inhibitory effect of tolvaptan on cell proliferation was progressively reduced when cells were exposed to low [Na^+^] and it was completely blunted at the lowest [Na^+^] (90 mM) in all cell lines (Fig. 2C).

**Fig. 3:**
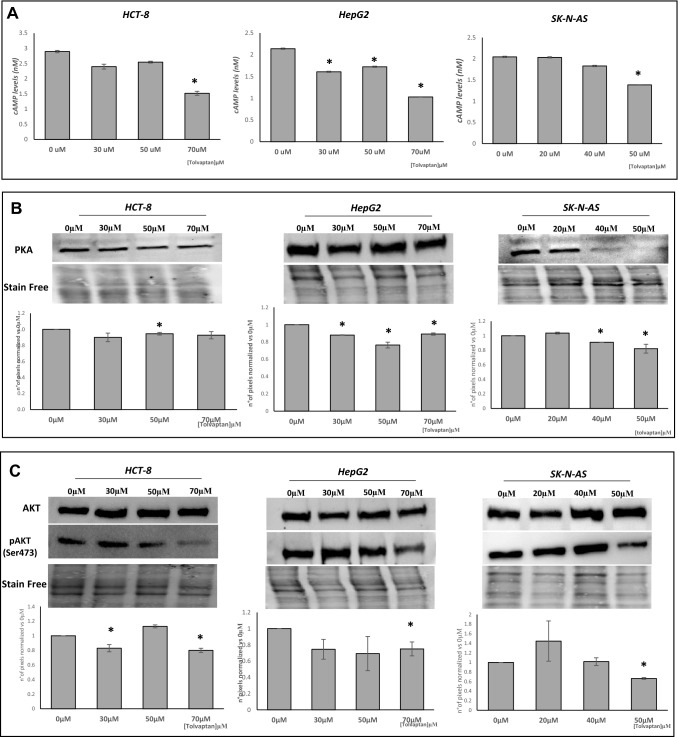
Effect of tolvaptan on cAMP, PKA and AKT/pAKT. Elisa assay of cAMP levels (**A**), and Western Blot analysis of PKA (**B**), AKT and Ser473-phosphorylated AKT (pAKT) (**C**). Images are representative of three different experiments, and graphs show cAMP, PKA and pAKT pixels /AKT pixels normalized *vs* 0 µM tolvaptan (*p ≤ 0.05)

PKA is the first key regulator of the V_2_ receptor signalling pathway. Therefore, the effect of tolvaptan on cAMP levels was determined and was found to be reduced in all cell lines (Fig. 3A). Accordingly, the expression of the catalytic α subunit of PKA was reduced in cells exposed to tolvaptan. (Fig. 3B). The PI3K/AKT pathway was also investigated, and a reduced pAKT/AKT ratio was observed upon tolvaptan treatment (Fig. 3C).

### Cell cycle and apoptosis

**Fig. 4:**
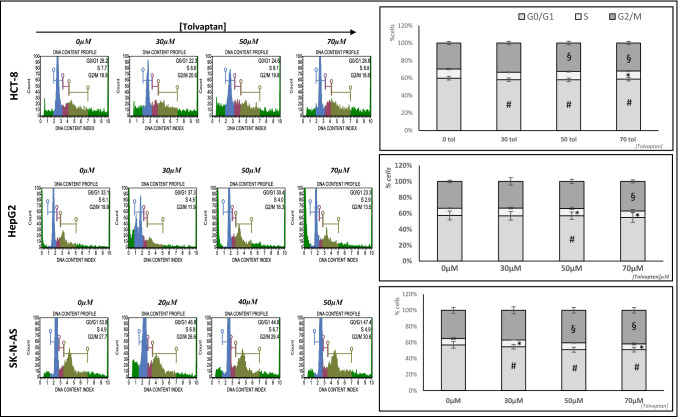
Effect of tolvaptan on cell cycle progression. Cytofluorimetric analysis of cell cycle; plots are representative of cell cycle trend and indicate % of G0/G1, S and G2/M cells. Graphs show the results from three experiments and are expressed as % of cells (mean ± SE), normalized *vs* 0 µM tolvaptan. (# = G0/G1 p ≤ 0.05 *vs* 0 µM; * = S p ≤ 0.05 *vs* 0 µM; § = G2/M p ≤ 0.05 *vs* 0 µM)

The effect of tolvaptan on cell cycle progression was analyzed. The number of cells in G0/G1 dose-dependently decreased upon the exposure to increasing concentrations of the drug, in all cell lines tested. Similarly, tolvaptan induced a decrease in the number of cells in S phase, whereas cells in G2-M appeared increased (Fig. 4).

**Fig. 5:**
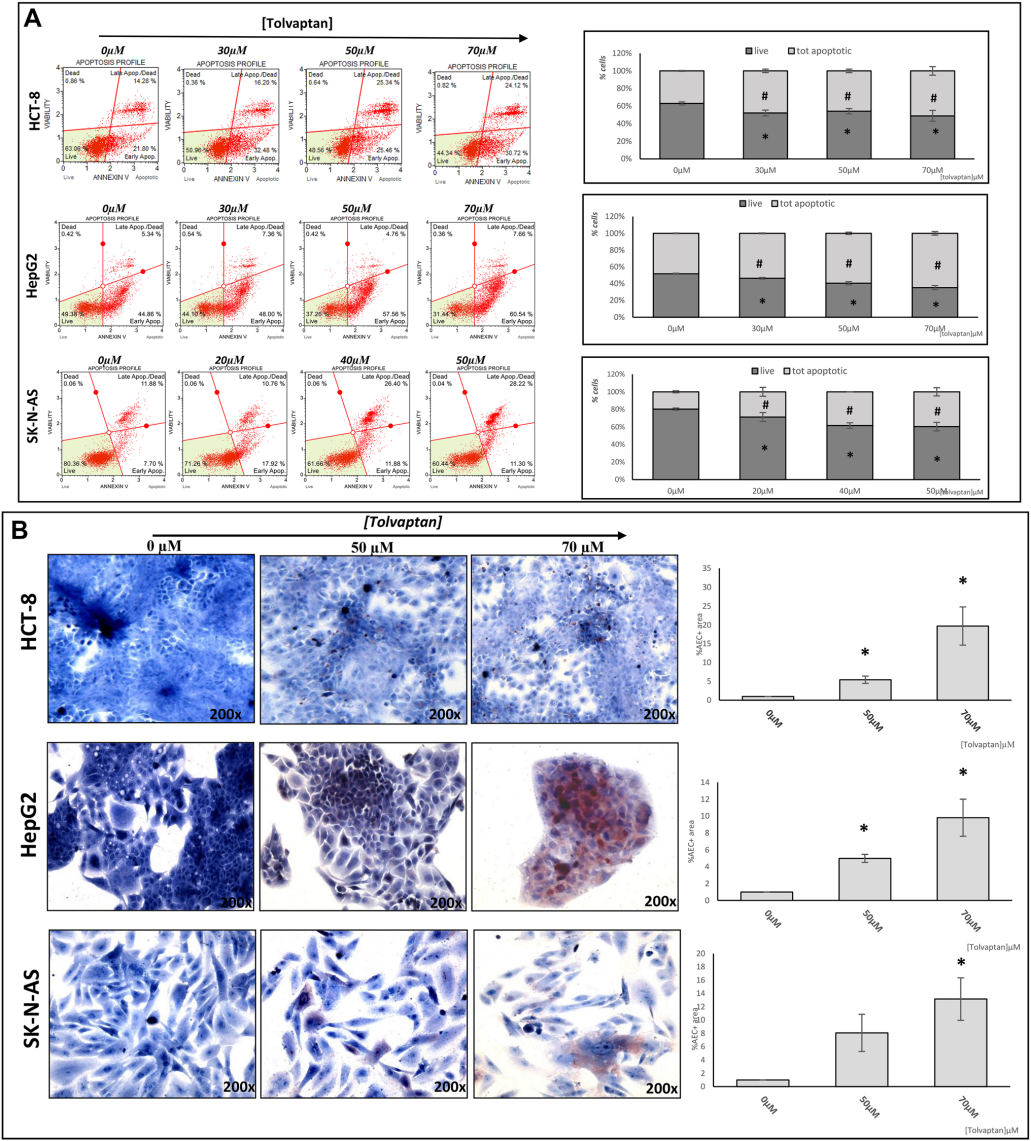
Effect of tolvaptan on cell death. The effects of tolvaptan on apoptosis were assessed by cytofluorimetry and results are expressed as mean ± SE of the % of live and dead cells from three different experiments (# = apoptotic cells p ≤ 0.05 *vs* 0 µM; * = live cells p ≤ 0.05 *vs* 0 µM) (**A**). Immunocytochemistry of caspase-3 on cancer cells at increasing doses of tolvaptan. Tot apoptotic = total apoptotic cells (early and late) (**B**); images are representative of two different experiments, and graphs show the % of positive cells normalized vs 0 µM tolvaptan (*p ≤ 0.05)

With regard to apoptosis, the exposure to the V_2_ receptor antagonist was associated with an increase in the percentage of apoptotic cells in HCT-8, HepG2 and SK-N-AS cells, together with a decrease of live cells (Fig. 5A). This finding was paralleled by an increased immunostaining for caspase-3 (Fig. 5B).

### Anchorage-independent growth and invasivity

**Fig. 6:**
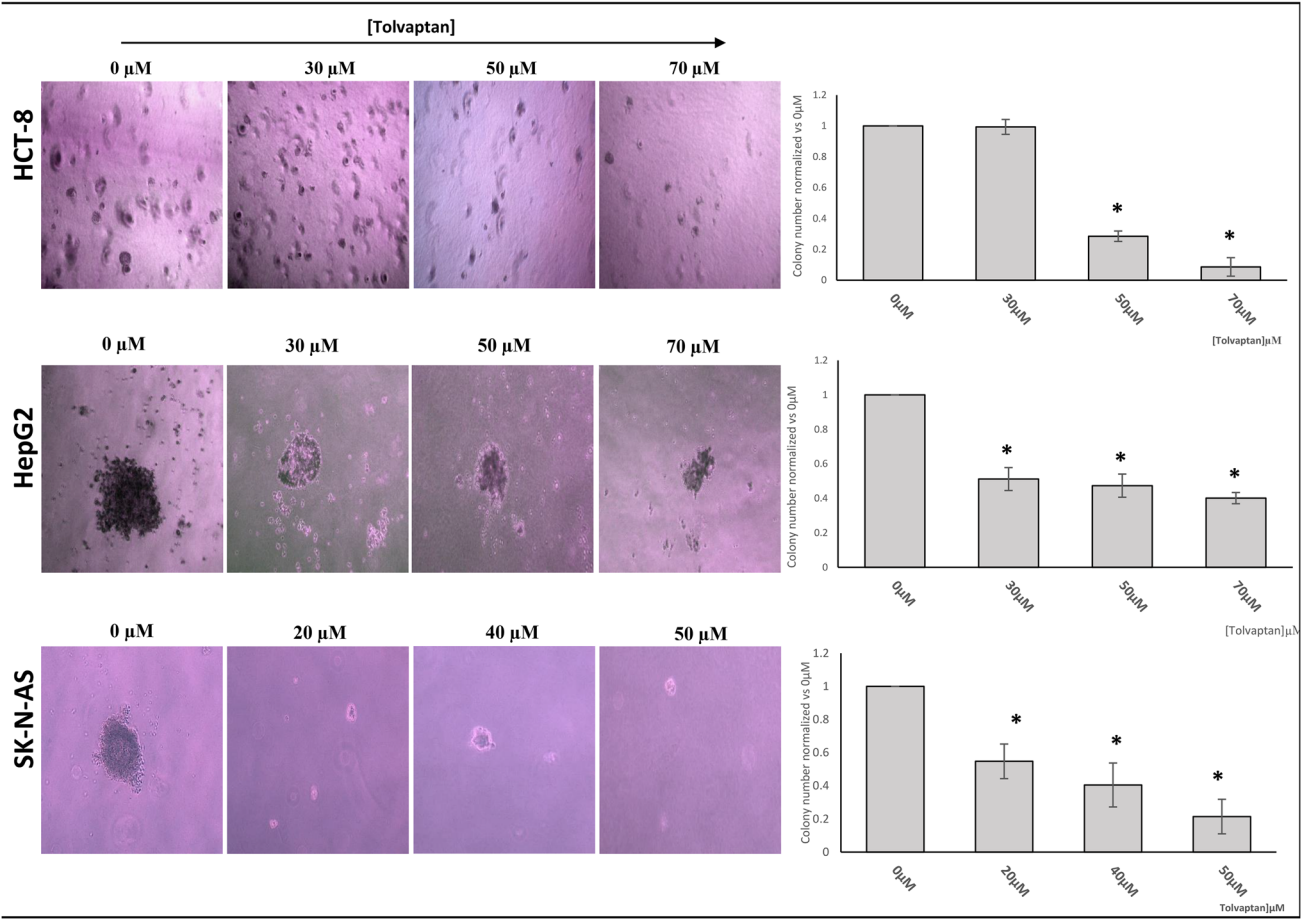
Effect of tolvaptan on anchorage-independent growth. Cells were grown for three weeks in soft agar. Images are representatives of colony formation (magnification 100X), and graphs represent the average number of colonies in eight different fields from three independent experiments. Results are expressed as mean ± SE. (*p ≤ 0.05 *vs* 0 μM tolvaptan)

The ability to grow in the absence of a solid substrate is a well-known feature of cancer cells in vitro. HCT-8, HepG2 and SK-N-AS cells proliferate and form colonies in soft agar [27, 40]. We demonstrated that the exposure to tolvaptan effectively reduced the number of cell colonies able to grow in soft agar (Fig. 6).

**Fig. 7:**
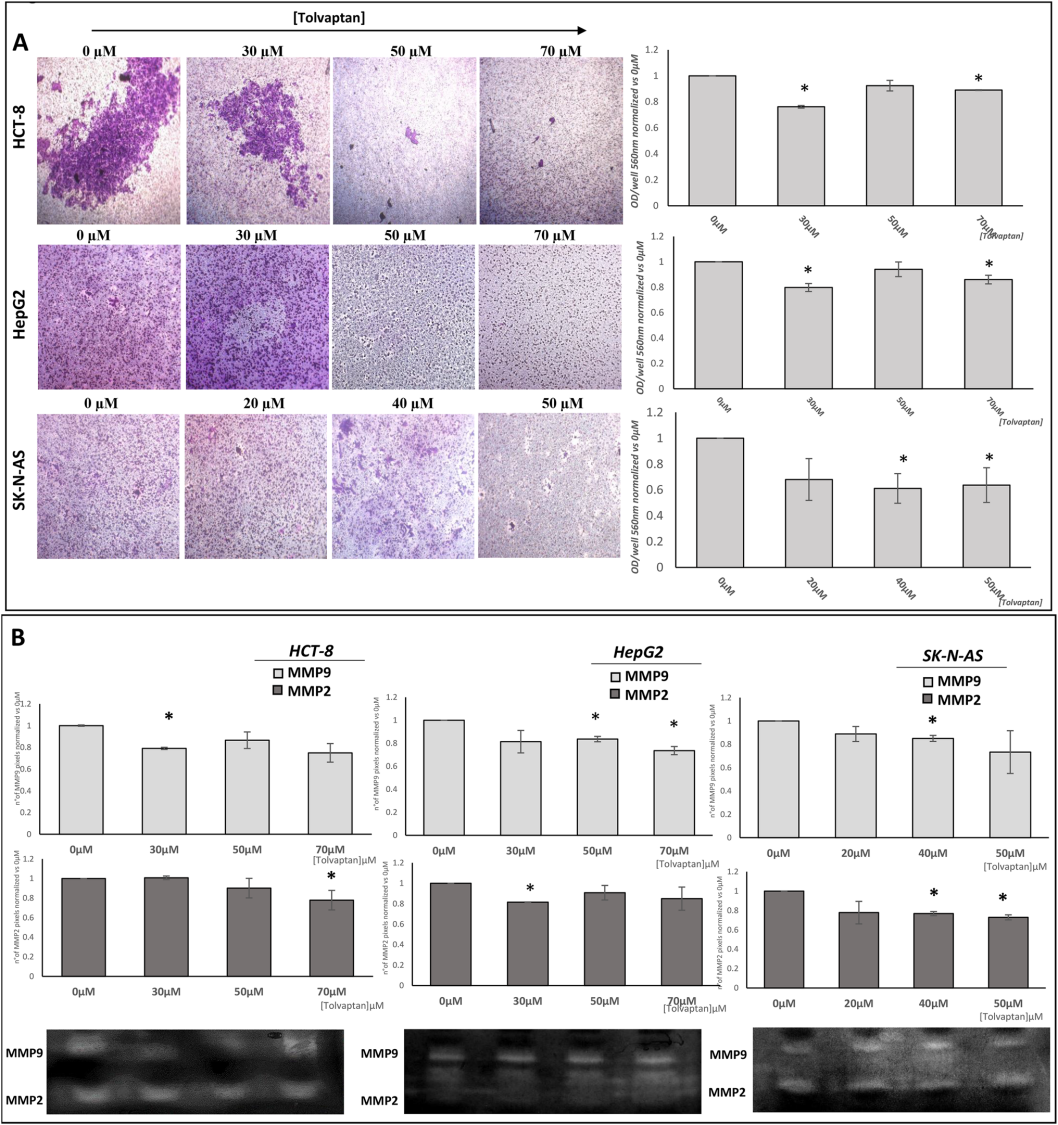
Effect of tolvaptan on invasion. Cell invasion was analyzed through matrigel-coated invasion chambers; images are representative of invading cells after crystal violet stain, and graphs show the median value from three different experiments normalized *vs* 0 µM tolvaptan.(*p ≤ 0.05) (**A**). The zymography assay was used to analyze the type IV collagenases activity (MMP2 and MMP9) by tumour cells after tolvaptan treatment. Images are representative of the areas of gels degradation by metalloproteinases, and bars represent the mathematical ratio of pixels from three experiments normalized *vs* 0 µM tolvaptan (*p ≤ 0.05) (**B**)

In contrast with the observation that the invasiveness of cancer cells is amplified in low extracellular [Na^+^] [27, 39], and in agreement with previous observations in small cell lung cancer H69 cells [39], tolvaptan blunted the ability of HCT-8, HepG2 and SK-N-AS cells to cross matrigel-coated membranes (Fig. 7A).

Zymography assays indicated that impaired cell invasiveness induced by tolvaptan was associated with the reduction of type IV collagenases activity (MMP2 and MMP9) (Fig. 7B).

### RhoA/ROCK1-2 pathway

The RhoA/ROCK1-2 pathway has an important role in the regulation of cell movement via the remodeling of actin cytoskeleton. Previous evidence indicated that low extracellular [Na^+^] activates this pathway [27, 39]. In addition, we have shown that tolvaptan has an inhibitory effect in H69 cells [39].

**Fig. 8:**
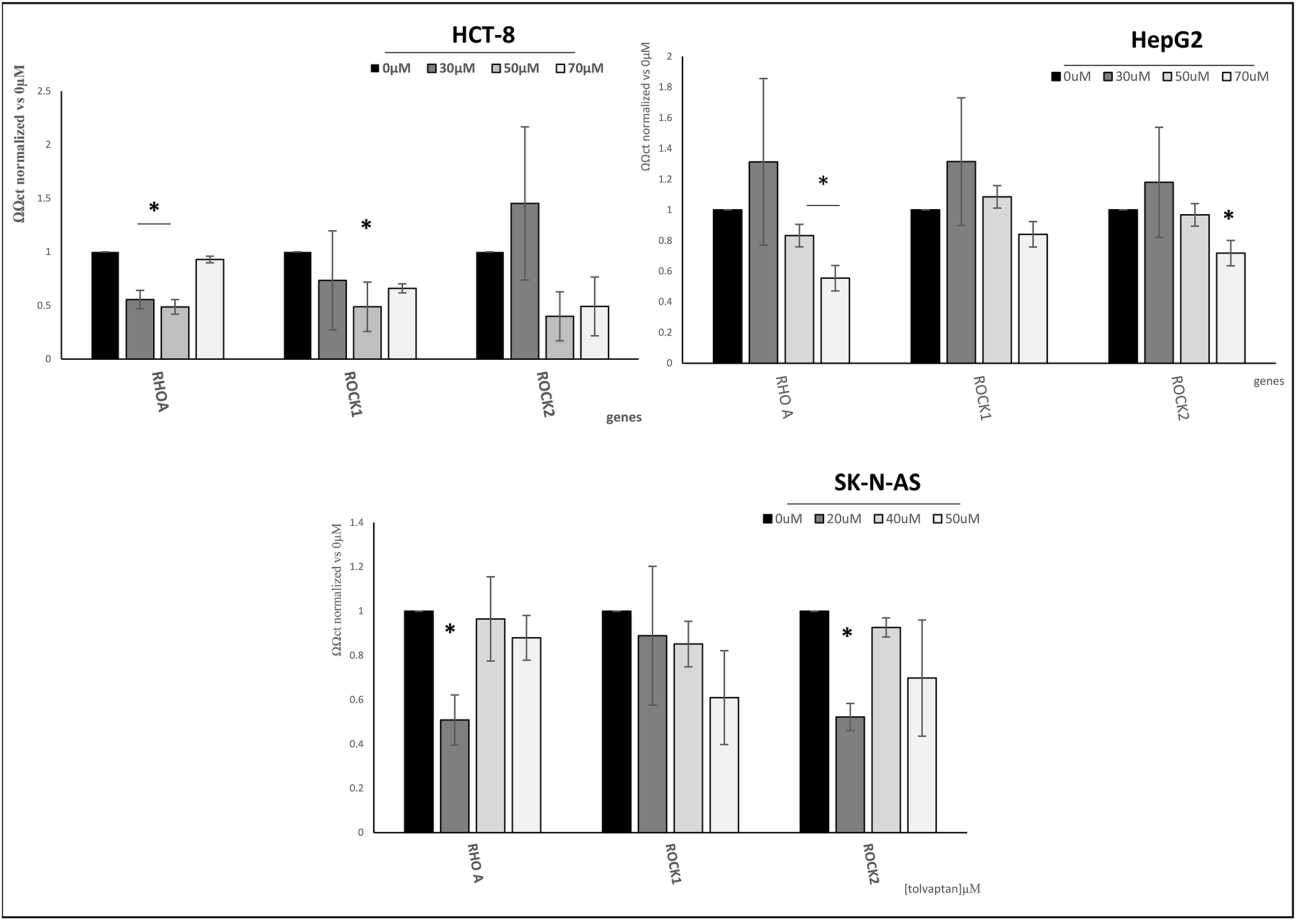
Tolvaptan regulation of RhoA, ROCK1 and ROCK2 mRNA. Real Time qPCR analysis of the amount of RhoA, ROCK1 and ROCK1 mRNA upon tolvaptan exposure. Results are expressed as ΔΔct normalized *vs* 18S, used as the housekeeping gene. Bars represent the mean ± SE from three different experiments, normalized *vs* 0 µM tolvaptan (*p ≤ 0.05)

**Fig. 9:**
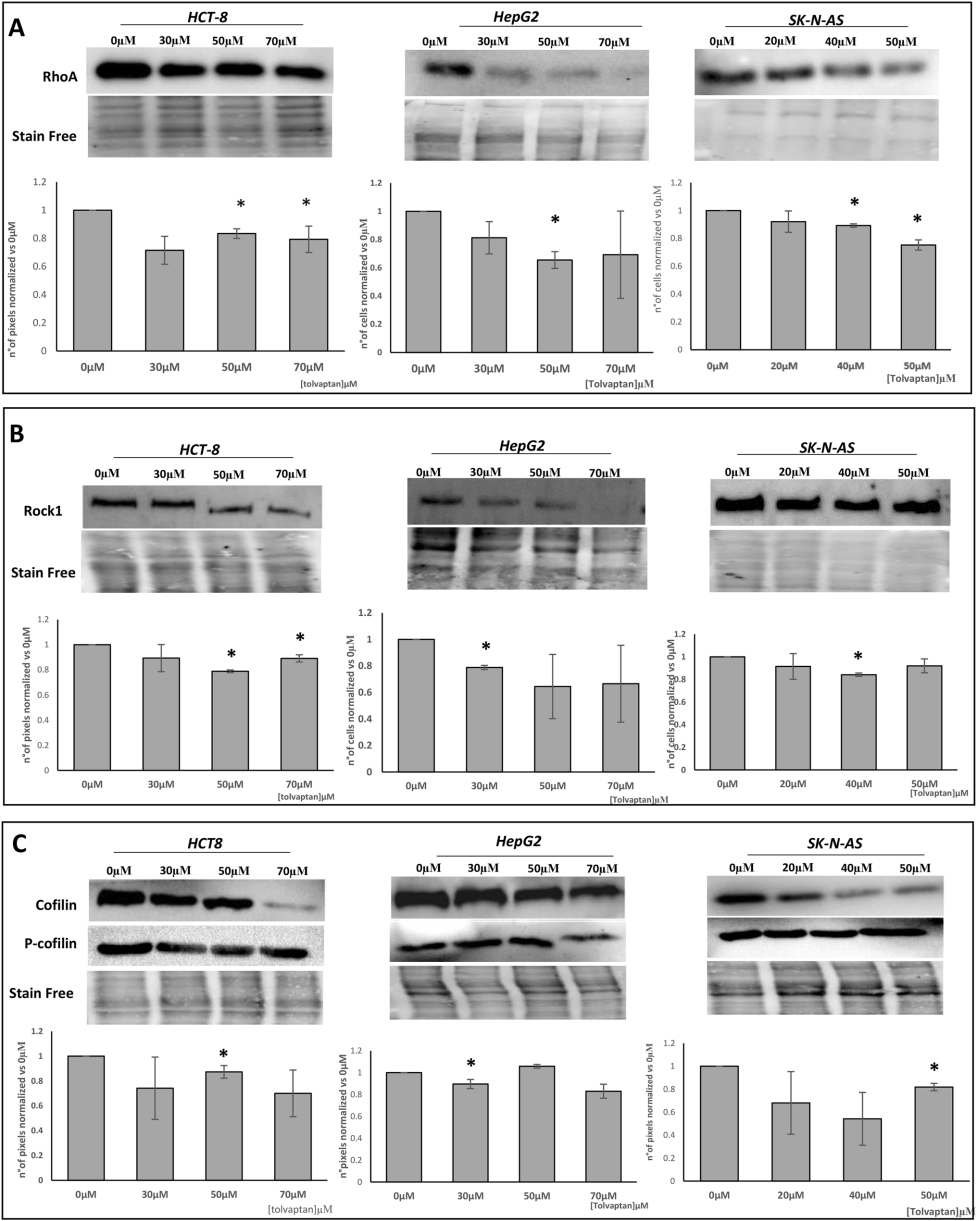
Tolvaptan regulation of RhoA/ROCK1-2 pathway. RhoA (**A**), ROCK1 (**B**), and cofilin/P-cofilin (Ser3) (**C**) protein expression were analyzed by Western blot and images show representative gels from three different experiments. The plots represent the ratio between protein/stain free pixels and results are expressed as mean ± SE normalized *vs* 0 µM tolvaptan. (*p ≤ 0.05)

Here, we found that tolvaptan reduced the mRNA amount of RhoA, ROCK1, ROCK2 in HCT-8, HepG2 and SK-N-AS cells (Fig. 8). These data were confirmed by the analysis of protein expression by Western blot analysis, (Fig. 9A and B). Overall, the degree of the inhibitory effect of different concentrations of tolvaptan on RhoA/ROCK1-2 expression varied in the different cell lines and significant differences were more often observed in HCT-8 cells. The downstream effect of the inhibition of the RhoA/ROCK1-2 pathway was confirmed by the reduced cofilin/P cofilin ratio upon tolvaptan exposure (Fig. 9C), similarly to what had been previously observed in H69 cells [39].

## Discussion

In recent years it became clear that hyponatremia may have a negative impact on the prognosis of cancer patients. Recent evidence indicated that the V_2_ receptor antagonist tolvaptan has antiproliferative effects in vitro in cells from kidney, liver and small-cell lung cancer [37–39].

In this study, we evaluated the effect of tolvaptan in different cell lines from colon cancer (HCT-8), hepatocarcinoma (HepG2) and neuroblastoma (SK-N-AS). No previous study had assessed the effect of this molecule in colon cancer or in neuroblastoma. HepG2 cells had been used in a previous study [37] and have been added here in order to have a validated control. In addition, hyponatremia likely due to ectopic AVP secretion has been described, yet rarely, in colon cancer [41], hepatocarcinoma [42] and neuroblastoma [43].

First, we assessed the presence of the V_2_ receptor in the selected cell lines. This receptor is normally located on the basolateral surface of the cells of renal collecting tubules and it is activated by AVP binding, which leads to cAMP generation, PKA activation and then to the synthesis and activation of the aquaporin 2 water channel [29]. We found that the three cancer cell lines express the V_2_ receptor, which is located in the cell membrane. Tolvaptan effectively inhibited cell proliferation with ICs_50_ in the micromolar range, similarly to data previously reported in the literature [37–39]. In particular, the IC_50_ for HCT-8 cells was 52 µM, for HepG2 38 µM and for SK-N-AS 40 µM. Accordingly, the amount of cAMP and the expression of the catalytic α subunit of PKA appeared to be reduced upon tolvaptan treatment, as well as the pAKT/AKT ratio, in all the three cell lines.

Interestingly, the anti-proliferative effect of tolvaptan was markedly reduced, yet not abolished, with the exclusion of the lowest [Na^+^] (i.e. 90 mM), in cells exposed to low [Na^+^]. This result is in keeping with previous experimental data that showed that low [Na^+^] promotes tumoural cell proliferation, including HCT-8 and SK-N-AS cells, that were included in the present study [27, 39].

Then, we analyzed the effect of tolvaptan on cell cycle progression. The number of G0-G1 and S cells decreased upon tolvaptan exposure in all the three cell lines, whereas cells in G2-M appeared increased. These findings are in keeping with similar published observations [37, 38] and suggest that tolvaptan prevents cell cycle progression toward cell division.

On the other hand, when we analyzed the effect of tolvaptan on apoptosis by citofluorimetry, we observed a significantly increased number of apoptotic cells at all the drug concentrations that were used and in all cell lines. Accordingly, the number of live cells decreased. In agreement with these data, we also found that the amount of expression of caspase-3, a key modulator of apoptosis, increased upon tolvaptan exposure. The effect of tolvaptan in promoting apoptosis observed here is in agreement with previous findings in small cell lung cancer cells [39], and confirm the data obtained in HepG2 cells [37].

It is known that growth in the absence of a solid support is a feature of malignant cells, and this has been shown previously in HCT-8, HepG2 and SK-N-AS cells [27, 40], in which low [Na^+^] was associated with an increased number of colonies. Here, we demonstrated that tolvaptan effectively reduced the ability of these cells to form colonies, when grown in soft agar, similarly to our previous results in small cell lung cancer cells [39].

Finally, we observed that the invasiveness of these cells was also counteracted by tolvaptan, and that this effect was associated with a reduced activity of type IV collagenases. In agreement with this finding, we observed that the RhoA/ROCK1-2 pathway was inhibited. This pathway is involved in the regulation of cytoskeleton dynamics, and thus in cell movement. In cancer, it has an important role in the induction of the metastatic cascade [44]. Interestingly, high levels of ROCK1 expression have been associated with a worse prognosis in several tumours, such as neuroblastoma, bladder, laryngeal and breast cancer [45–48]. RhoA overexpression has been associated with a significantly reduced disease-free and distant metastasis-free survival in cervical squamous cell carcinoma [49]. A facilitatory role for RhoA in inducing a more aggressive phenotype has been observed also in other malignancies, such as for instance lung adenocarcinoma [50], ovarian carcinoma [51] and lymphoma [52].

Accordingly, the RhoA/ROCK1-2 pathway has been suggested as a possible therapeutic target in cancer [45, 53, 54].

With regard to the downstream effector cofilin, we observed that tolvaptan determined a reduced cofilin/P cofilin ratio in all the three cell lines. Cofilin depolymerizes F-actin and creates new actin monomers for polymerization, thus accelerating actin turnover and modulating cell movement. Upon phosphorylation, cofilin is inactivated [55, 56].

Overall, these findings add new evidence to the few data reported in the literature, so far, in favour of an antitumoural effect of the V_2_ receptor antagonist tolvaptan. Noticeably, the serum peak concentration of tolvaptan after a single dose of 30 mg in healthy volunteers is in the low micromolar range [57]. In ADPKD the daily regimen of tolvaptan may reach 120 mg [58]. These concentrations are very similar to those that effectively inhibited cell proliferation and invasion in vitro in our experimental models.

Vaptans have opened a new era in the treatment of hyponatremia secondary to SIAD and have been the first non-peptide molecules that targeted AVP receptors [29]. As already mentioned, in the past decade it became evident that hyponatremia negatively affects the course of different pathologies, including cancer. Remarkably, [Na^+^] correction has a favourable effect on patients’ outcome [23, 59–61]. However, vaptans appear to have an additional and direct antitumoural effect, which recalls their mode of action in reducing the growth of cysts in ADPKD [36, 62, 63]. AVP receptors are expressed in different cancer types, including breast, pancreatic, colorectal, gastrointestinal cancer, and small cell lung carcinoma [64, 65]. V_1_ receptor activation has been associated with increased cell growth [66, 67], whereas a more controversial role has been associated with the V_2_ receptor. Nevertheless, a proliferative effect related to V_2_ receptor activation has been observed in renal carcinoma cells [68]. The recent findings on the antitumoural effects of tolvaptan, a selective V_2_ receptor antagonist [39, 69, 70], in addition to the data shown in the present study, suggest that the V_2_ receptor can be considered a possible target against cancer. This hypothesis needs to be further confirmed in *in* vivo studies. With regard to this point, one study has demonstrated that tolvaptan reduced cell proliferation and angiogenesis, while increasing apoptosis, in a mouse xenograft model of renal cancer [38].
